# Physiological and psychological recovery in two pure forests: interaction between perception methods and perception durations

**DOI:** 10.3389/fpubh.2024.1296714

**Published:** 2024-04-23

**Authors:** Xiaogang Sun, Qinglan Li, Xin Zhang, Miao Sun, Jiahui Yin, Jingyi He, Yige Zhong, Wei Ning

**Affiliations:** ^1^College of Forestry and Grassland Science, Jilin Agricultural University, Changchun, China; ^2^Jilin Provincial Key Laboratory of Tree and Grass Genetics and Breeding, College of Forestry and Grassland Science, Jilin Agricultural University, Changchun, China

**Keywords:** forests bathing, physiological health, psychological health, perception methods, perception durations

## Abstract

The forest experience is good for people’s physical and mental health. However, few studies on the effects of pure forest based on the duration and way of experience on people’s physical and mental recovery. In this study, we took 180 first-year college students as research objects and conducted experiments in *Pinus sylvestris* and *Betula platyphylla* and the control group of grass plot. The changes of physiological and psychological indexes of the subjects were compared by two perception methods (onsite perception, video perception) and three perception duration (10 min, 20 min, 30 min). The results indicated that: (1) Differences between the two pure forests were mainly reflected in short-term recovery of diastolic blood pressure (DBP) and long-term recovery of total mood disorder (TMD). (2) Video perception was more conducive to short-term recovery of systolic blood pressure (SBP) and diastolic blood pressure (DBP). (3) Viewing the *Pinus sylvestris* for 20 min in different ways was the best way to relieve stress. It is suggested that, *Pinus sylvestris* can be used as the rehabilitation perception material, and reasonable path length or perception time can be selected for landscape construction in future. These results can provide scientific reference for landscape design based on forest health and environmental perception.

## 1 Introduction

With the progress and development of the times, the process of urbanization has shown an unprecedented dynamic trend. More than half of human beings live in urban areas (1). People encounter more and more psychological pressure from financial, family, occupational and other stressors (2–5). Increased stress may lead to a series of adverse reactions such as memory loss, attention loss, and insomnia (6–8), which affect people’s normal life and work, hinder people’s development, and even cause depression, diabetes, cancer, cardiac arrest and other diseases (9–12). The forest environment has been confirmed to have a positive impact on human physiological and psychological health, a short and leisurely forest trip, that is, “forest bathing,” can not only bring positive changes in physiological health for people living in urban environments, but also improve emotional anxiety and depression (13–15). Although scientific and technological progress has increased the way people perceive and experience nature, the opportunities and time for people to contact nature are still very limited, and the scientific and reliability of perception methods are still uncertain. The cross effects of perception method and perception durations is also inconclusive, which leads to the mismatch between the public’s demand for the natural environment and the actual landscape design (16). Therefore, it is necessary to study the scientific perceiving methods and the effective perceiving durations.

### 1.1 Perception of different forests

Studies have found that the natural environment can reduce stress, give people a sense of psychological satisfaction and stability, and even improve immunity and resist disease (17–20). Forests is an important part of the natural environment. The forests environment is naturally quiet. According to the theory of natural therapy, forests areas are beneficial to health and can relax the mood (21). Slow walking in the forest can relieve stress and bring mental stability (22, 23). Perception of forests landscape can improve mental state (24), help people free themselves from the pressure of daily living space (25–29). However, forests are not all the same, and the potential of forests to relieve stress cannot be evenly distributed among different forests (30). Sandro et al. confirmed in a study that coniferous forests have better stress relief for humans in winter (31). Wang et al. found that evergreen tree species have a significant effect on human psychological recovery in spring, autumn and winter (32). Different forests may lead to different restoration effects due to their vertical structure density, canopy closure and other structural differences within the forests (33–35). Therefore, it is of great significance to explore the effect of different forests on the physical and psychological recovery of human body for the construction of health forest with the goal of health care.

### 1.2 Perception in different methods

When people find that they can relieve stress and fatigue by contacting the forests environment, they begin to be more eager to perceive the forests landscape. However, the heavy pressure of work and life forces people to stay indoors for 70–90% of the day (36). Therefore, how to effectively perceive forests landscape has become a research hotspot in the field of forests therapy and environmental perception. Previous studies have shown that onsite perception and off-site perception are two main ways of landscape perception. Onsite perception means that the perception of subjects is affected by environmental factors and onsite landscape. Zhao et al. found that middle-aged and older adult people can obtain physiological and psychological benefits through onsite viewing of peony activities (37). Duan et al. have shown that onsite perception of plant communities has a greater healing effect on subjects than video appreciation of plant communities (38). Off-site perception means that the subject’s perception is completed indoors, mainly through visual perception images to achieve a healing effect, basically not affected by environmental factors. Wang et al. found that watching a neat bamboo shrub video can have a more beneficial physical and mental impact on the human body (39). Duan et al. showed that the use of perceptual plant communities alternately or superimposed with videos and photos can maximize the healing effect (40). Although the two perception methods have been widely used and studied, it is necessary to explore the differences between the perception methods of different forests because the research results of landscape perception are inconsistent, and there are few perception studies based on different forests.

### 1.3 Perception in different durations

The effect of landscape perception is not only affected by the way of perception, but also varies with the length of perception. Jo’s research showed that exposure to a green environment will have an immediate effect on physical and mental recovery, self-esteem and emotions show the greatest changes within 5 min (41). Ning et al.’s research showed that 5 min of short-term environmental exposure is beneficial to the physical and mental recovery of young people (42). A field experiment in Japan also proved that 15 min of observation and walking in the forest can induce physiological relaxation (43). An Australian study had shown that people who spend 30 min or more in the green space have a lower probability of depression and hypertension (44). Although studies had shown that long-term environmental exposure still has a positive effect on human health recovery, most of them are based on the periodic accumulation of short-term recovery effects (45–47). Therefore, the short-term perception of green environment on the physical and mental recovery benefits of visitors cannot be ignored, but the optimal time dose of short-term perception remains to be studied.

In view of this, this study completed the relationship between landscape perception and public health benefits of two pure forests (*Pinus sylvestris*, *Betula platyphylla*) and a control group (grass plot) by means of onsite perception and video perception, explored the differences in short-term physiological and psychological recovery based on different time doses, to provide scientific basis for future landscape perception and forest health evaluation. Our research aims to address the following issues:

(1) Are there differences of two pure forests on the recovery of physiological and psychological indicators?
(2) How do the perceiving methods affect the subjects’ physiological and psychological recovery?
(3) How does the interaction of perception methods and perception durations affect the physiological and psychological recovery of subjects?

## 2 Materials and methods

### 2.1 Sample space selection

**Figure 1:**
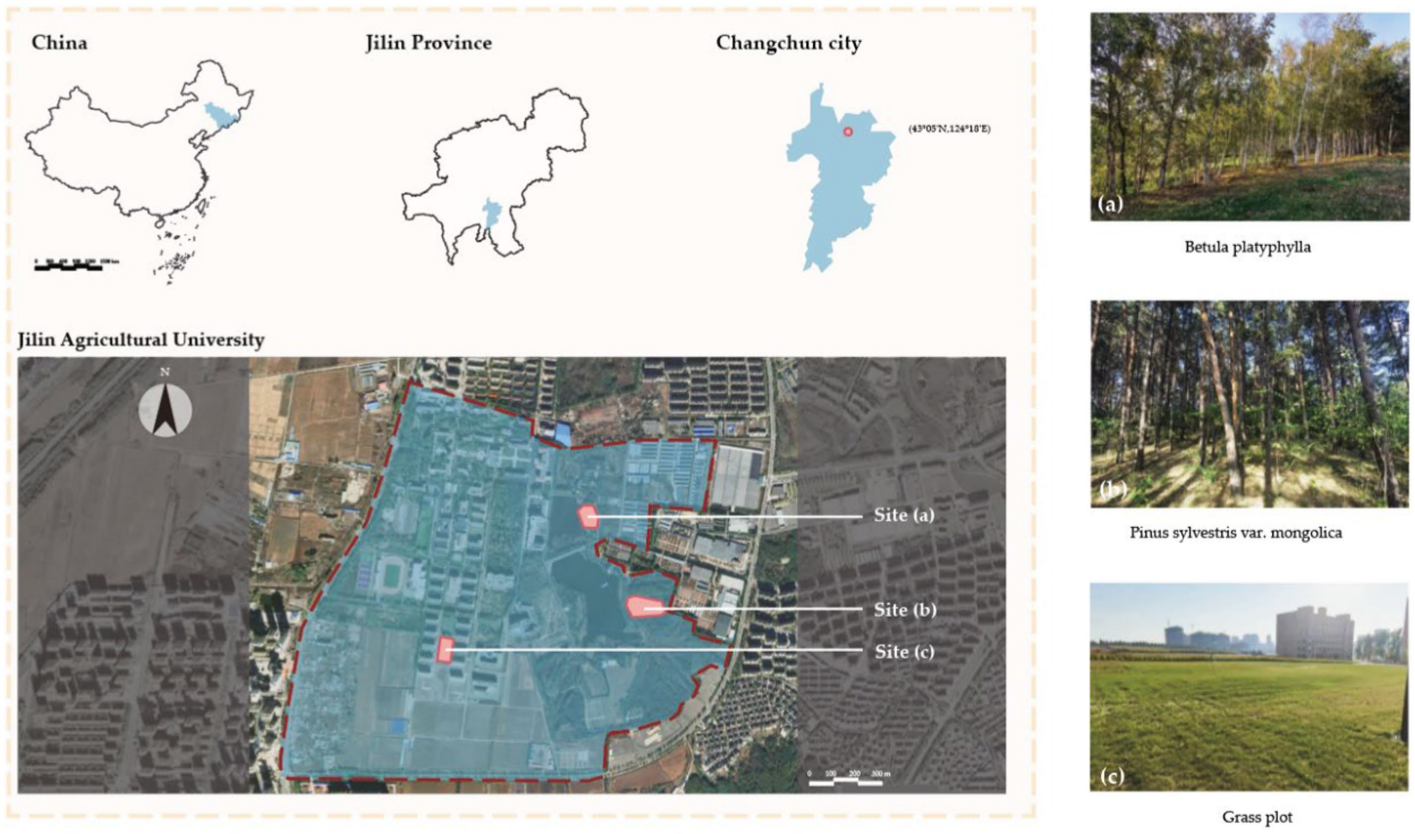
Map of experimental location and study area. **(A)** BP, *Betula platyphylla*. **(B)** PS, *Pinus sylvestris*. **(C)** GP, Grass plot.

Forests areas of China ranks first among 48 Asian countries (48), and pure forests are abundant. In this study, we focus on pure forests with high ornamental value that widely found in the Northeast China. The experimental plot is located on the campus of Jilin Agricultural University (43°51′N, 125°18′E) in Changchun City, Jilin Province, Northeast China. Previous studies have shown that understory space is more popular with visitors (49). Therefore, in the sample selection, we chose the *Pinus sylvestris* forests (PS), the *Betula platyphylla* forests (BP) and the Grass plot (GP) as the control group for study (Figure 1).

In this study, we selected two stimulation methods, namely, onsite perception and video perception. Both onsite perception and video perception experiments were completed between 1 October 2022 and 7 October 2022. The onsite perception test was carried out in sunny weather and at a suitable temperature. Before the test, non-testers in the site were emptied, and the distance between the test area and the forest edge was ensured to be 8–10 meters, thereby reducing the interference of external factors. The video perception test material was filmed by the EOS70D Canon camera, which is carried out at 8:00–12:00 with sufficient light and sunny weather. To obtain a better immersive feeling for later viewing, two shooting methods are adopted: tripod fixed shooting and pan-tilt assisted walking shooting. A total of 16 videos were taken by the five-point sampling method. Under the guidance and advice of landscape design experts, these videos were edited, and finally the three spatial videos required for the experiment were selected for this experiment.

### 2.2 Selecting subjects and grouping

**Figure 2:**
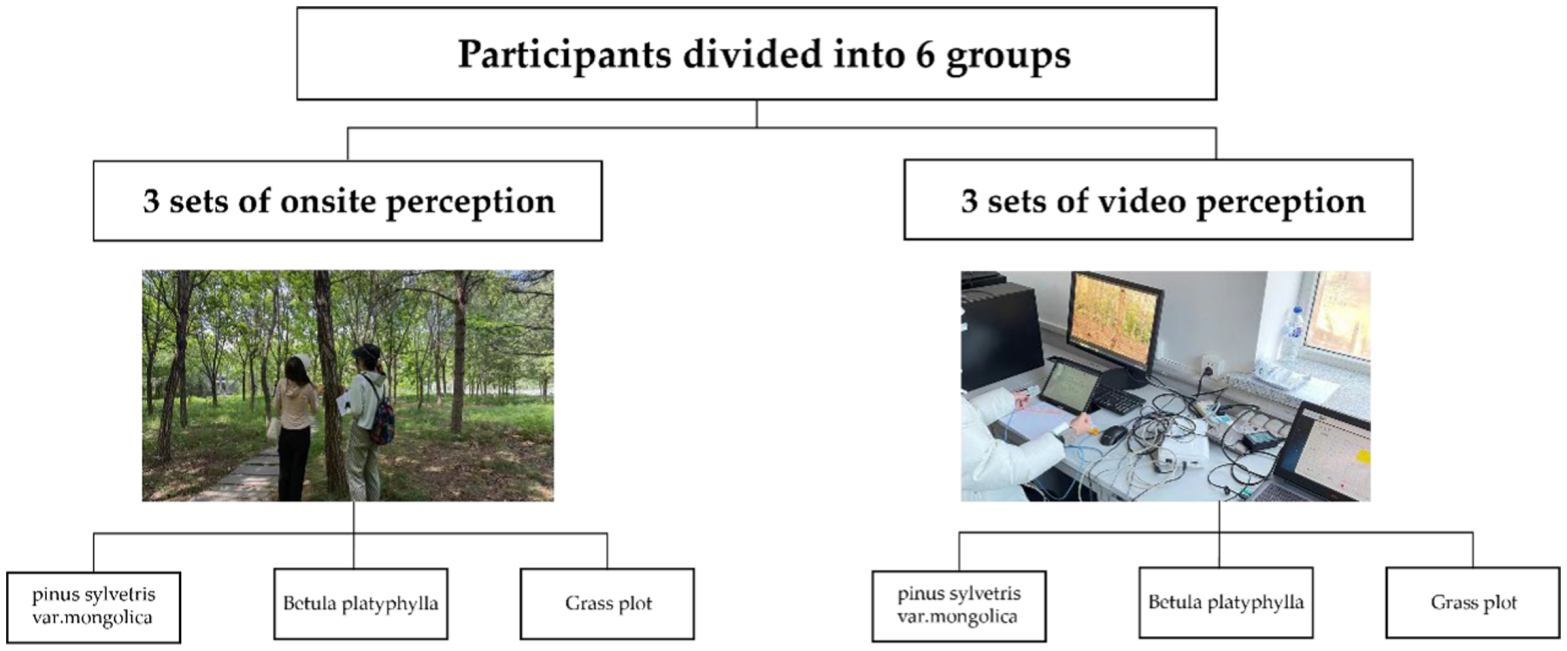
Grouping arrangement and test scenario.

The subjects recruited in this experiment were first-year students from the College of Forestry and Grassland Science of Jilin Agricultural University. The freshmen are in a certain unknown state of the campus environment, which can minimize the experimental errors caused by familiarity with the experimental plots and exclude patients with heart disease or other medical history. Subjects were also told to forbid any strenuous physical activity, smoking, drinking throughout the experiment, before and during the experiment, who disagreed or did not want to continue participating in the experiment was excluded. All procedures in this study are consistent with the “Declaration of Helsinki.” The experiment initially recruited 185 participants, of which five participants failed to participate in the whole experiment due to the conflict of student community activities. A total of 180 subjects participated in the experiment. During the experiment, 180 subjects were randomly divided into six groups, which are“Onsite perception *Pinus sylvestris* forest (OPS), Video perception *Pinus sylvestris* forest (VPS), Onsite perception *Betula platyphylla* forest (OBP), Video perception *Betula platyphylla* forest (VBP), Onsite perception Grass plot (OGP) and Video perception Grass plot (VGP),” 30 subjects in each group, and 30 subjects were randomly divided into 3 teams, 10 subjects in each team, the experiments are organized in teams (Figure 2). In addition, through interviews and physiological and psychological indicators tests, we confirmed that the subjects were under the pressure of the new living environment and learning courses, and the initial values of the physiological indicators of each group were not significantly different through analysis of variance (Table 1).

**Table 1:** Basic information of different groups in this study (*n* = 180).

Groups	Age	SBP	DBP	HR	TMD
OPS	18.57 ± 0.94	108.03 ± 7.45	72.17 ± 6.05	73.10 ± 10.25	−18.43 ± 6.72
VPS	18.37 ± 0.76	109.70 ± 10.59	72.40 ± 6.80	74.93 ± 10.26	−18.37 ± 7.07
OBP	18.53 ± 0.94	107.00 ± 9.90	70.30 ± 8.52	77.57 ± 10.30	−17.07 ± 6.95
VBP	18.40 ± 0.77	106.30 ± 14.63	71.87 ± 9.31	78.17 ± 11.11	−17.20 ± 7.27
OGP	18.57 ± 0.90	110.63 ± 10.57	73.60 ± 8.32	77.43 ± 7.82	−17.53 ± 6.89
VGP	18.37 ± 0.81	108.27 ± 12.82	73.43 ± 10.41	76.77 ± 9.72	−17.87 ± 7.32
*p*	0.848	0.679241	0.687319	0.463648	0.961

SBP, Systolic blood pressure; DBP, Diastolic blood pressure; HR, Heart rate; TMD, total mood disorder (**p < 0.01, *p < 0.05).

### 2.3 Experimental design

#### 2.3.1 Test introduction

We designed a random experiment to study the effects of two pure forests and control group on the physiological and psychological recovery of the subjects through the interaction between two perception methods (Onsite perception, Video perception) and three perception durations (10 min, 20 min, 30 min). Each group was randomly assigned to one of the six scenarios. Only one onsite perception team and one video perception team were tested within a single test day (Figure 2). Subjects were subjected to a stress test before entering the test plot or sensing video to induce mental stress, then experienced and relaxed in each scenery. To evaluate and compare their physiological and psychological changes based on different perception durations, we tested the subjects’ SBP, DBP, HR during the experiment for physiological recovery every 10 min and used the POMS scale to test the situation of psychological recovery at the same time.

#### 2.3.2 Experimental process

##### 2.3.2.1 Onsite perception

We choose a sunny day with pleasant temperature from 1 October 2022 to 7 October 2022 as the measurement day. In the preparation stage, the subjects were guided to enter the vicinity of the sample site (isolated test environment), and the test process was described. When the subjects returned to calm, they tested the baseline of physiological indicators and filled in the first part of the psychological index questionnaire, and then pressured the subjects to complete 5 min national English level 6 listening test. During this process, the test instrument was continuously worn. After the pressure was applied, SBP, DBP, and HR were tested, and the second part of the POMS scale was filled in (P_pressure_). The subjects were guided to enter the sample environment to relax. The volunteers recorded the SBP, DBP and HR of the subjects every 10 min and guided the subjects to fill in the POMS scale again (P_10_, P_20_). After the onsite perception, the participants completed the third and last physiological index test and psychological scale filling (P_30_), and then removed the wrist sphygmomanometer, and the subjects left the test site.

##### 2.3.2.2 Video perception

The video perception test and the onsite perception test were carried out at the same time because the indoor temperature in October was more comfortable, and the indoor environment with normal light and no visual interference was selected. The subjects wore wrist band sphygmomanometers and explained the experimental procedures and precautions. The subjects were taken to the designated experimental site, and the subjects began to test physiological indicators and fill in the first part of the questionnaire after the sit-in was stable, and then the subjects were pressured before watching the video. The subjects were asked to complete 5 min national English level 6 listening test. After the pressure was applied, the SBP, DBP and HR were tested, and the second part of the POMS scale psychological questionnaire was filled out (P_pressure_). Volunteers began to play videos of the corresponding forest types, and the subjects watched the videos. During the 30 min viewing period, the volunteers recorded the SBP, DBP and HR of the volunteers every 10 min and guided the subjects to fill out the POMS scale again (P_10_, P_20_). After the subjects completed the third index test (P_30_), the experimental process was completed and the whole experiment was completed.

### 2.4 Measurement

#### 2.4.1 Physiological measures

Biofeedback measurement was used to measure physiological changes, including diastolic blood pressure (DBP), systolic blood pressure (SBP) and heart rate (HR). During the test, the Sean wrist electronic sphygmomanometer was selected to collect the physiological indexes of the subjects. To minimize the measurement error, three repeated measurements were performed during the experimental data collection of the subjects, and the average value of the data was finally taken.

#### 2.4.2 Psychological measures

The psychological test was based on the Chinese conventional POMS scale, and the reliability was between 0.60–0.82 (50). The scale is divided into three dimensions (Restlessness, Interested, Relaxed) and including 12 adjectives (Interest, Refresh, Energy, Vigor, Relax, Happy, Quiet, Calm, Insecure, Confusion, Sullen, Worry) describing mood and emotion as secondary indicators. From “not at all” to “very” rating scale, the subjects independently choose to describe their current emotions. Total mood disorder (TMD) is calculated according to the above three dimensions, namely TMD = Restlessness (Insecure + Confusion + Sullen + Worry) - Interested (Interest + Refresh + Energy + Vigor) – Relaxed (Relax + Happy + Quiet + Calm). The higher the value is, the worse the mental health is, and vice versa.

### 2.5 Data analysis

The agreement and data collection for this study were approved by Jilin Agricultural University Ethical Committee. All data were processed by SPSS 26.0 (IMB SPSS Statistics). Continuous data conforming to normal distribution or approximate normal distribution were statistically described by mean and standard deviation. The timeline is divided into 3 parts:


$$\Delta P_{10}=P_{\mathrm{pressure}}-P_{10}$$

$$\Delta P_{20}=P_{\mathrm{pressure}}-P_{20}$$

$$\Delta P_{30}=P_{\mathrm{pressure}}-P_{30}$$

In the formula, P is the mean value of each index, P_pressure_ is the mean value of each index after pressure application, P_10_ is the mean value of each index after 10 min of recovery, P_20_ is the mean value of each index after 20 min of recovery, P_30_ is the mean value of each index after 30 min of recovery, △P is the change value of each index, P_10_ is the change value of each index after 10 min of recovery, P_20_ is the change value of each index after 20 min of recovery, P_30_ is the change value of each index after 30 min of recovery. When △P is negative, it indicates that the index after different recovery time is greater than the index after pressure. When △P is positive, it indicates that the index after different recovery time is less than the index after pressure.

One-way ANOVA was used to compare the differences between different forests. Paired Sample T Test was used to compare the changes of physiological and psychological indicators under different perception methods. One-way ANOVA and Two-ways repeated-measures ANOVA were used to compare the differences between different groups under the interaction of perception methods and perception durations. *p* < 0.05 was considered statistically significant in all comparisons.

## 3 Results

### 3.1 Effects of forests on health recovery

After visitors perceive different forests and grass plot, the recovery amounts of DBP and TMD is significantly different (Table 2). Further analysis shows that the perception of PS is the most effective for alleviating the DBP of subjects. The short-term healing effect of DBP on GP is better than that of BP. The TMD recovery amounts of PS and BP were significantly greater than GP. With the increase of perception time, the TMD recovery amounts of PS decreased, while that of BP was the opposite. The results show that PS has obvious advantages in the recovery of DBP and TMD, GP for the recovery of DBP is also very good, BP for the recovery of TMD showed potential.

**Table 2:** Effects of forests on physiological and psychological indicators.

Indicator		PS	BP	GP	*p*
SBP	△P_10_	7.20 ± 5.77	5.63 ± 8.03	6.53 ± 6.25	0.445
	△P_20_	5.80 ± 6.83	6.17 ± 8.47	4.82 ± 8.77	0.639
	△P_30_	7.65 ± 7.27	6.23 ± 8.03	5.22 ± 8.18	0.235
DBP	△P_10_	8.05 ± 6.94	3.83 ± 6.87	5.33 ± 7.63	0.006**
	△P_20_	8.52 ± 7.29	4.12 ± 7.32	4.93 ± 7.44	0.003**
	△P_30_	4.70 ± 7.73	3.02 ± 7.07	3.98 ± 6.25	0.423
HR	△P_10_	5.83 ± 6.13	3.98 ± 7.17	4.15 ± 6.08	0.226
	△P_20_	5.22 ± 6.52	4.72 ± 5.54	5.02 ± 6.78	0.909
	△P_30_	4.47 ± 5.47	3.63 ± 6.59	3.15 ± 5.61	0.468
TMD	△P_10_	3.13 ± 3.24	3.92 ± 3.67	4.35 ± 3.43	0.150
	△P_20_	5.83 ± 3.95	3.83 ± 3.79	4.20 ± 3.95	0.013*
	△P_30_	5.40 ± 3.59	5.68 ± 3.44	3.93 ± 3.66	0.017*

PS, *Pinus sylvestris*; BP, *Betula platyphylla*; GP, Grass plot (**p < 0.01, *p < 0.05).

### 3.2 Effects of perception methods on health recovery

The analysis of the recovery of each indicator after the subjects perceived the two pure forests and grass plot showed that the perception methods had a significant effect on the short-term recovery of SBP and DBP. It had no significant effects on the short-term recovery and long-term recovery of HR and TMD (Table 3).

**Table 3:** Effects of perception methods on physiological and psychological indicators.

Indicator		Onsite perception	Video perception	*p*
SBP	△P_10_	4.79 ± 6.66	8.12 ± 6.45	0.001**
	△P_20_	4.58 ± 7.36	6.61 ± 8.59	0.090
	△P_30_	6.02 ± 7.79	6.71 ± 7.96	0.558
DBP	△P_10_	4.52 ± 7.10	6.96 ± 7.38	0.025*
	△P_20_	5.48 ± 7.48	6.23 ± 7.65	0.504
	△P_30_	3.90 ± 7.35	3.90 ± 6.75	1.000
HR	△P_10_	4.53 ± 6.49	4.78 ± 6.54	0.801
	△P_20_	4.97 ± 5.73	5.00 ± 6.80	0.972
	△P_30_	3.77 ± 5.52	3.73 ± 6.29	0.970
TMD	△P_10_	4.07 ± 3.41	3.53 ± 3.52	0.304
	△P_20_	4.09 ± 4.02	5.16 ± 3.87	0.071
	△P_30_	5.06 ± 3.76	4.96 ± 3.51	0.854

SBP, Systolic blood pressure; DBP, Diastolic blood pressure; HR, Heart rate; TMD, total mood disorder (**p < 0.01, *p < 0.05).

Further analysis found that the SBP recovery effect of subjects’ video perception of different forests was better than that of onsite perception of different forests. For the recovery of DBP, video perception was more conducive to the recovery of DBP. The results show that video perception is conducive to the short-term recovery of physiological health, the perception method has no significant difference on the long-term recovery of physiological health and psychological stress relief.

### 3.3 Effects of the interaction of perception methods and perception durations on health recovery

#### 3.3.1 Change of SBP indicator

**Figure 3:**
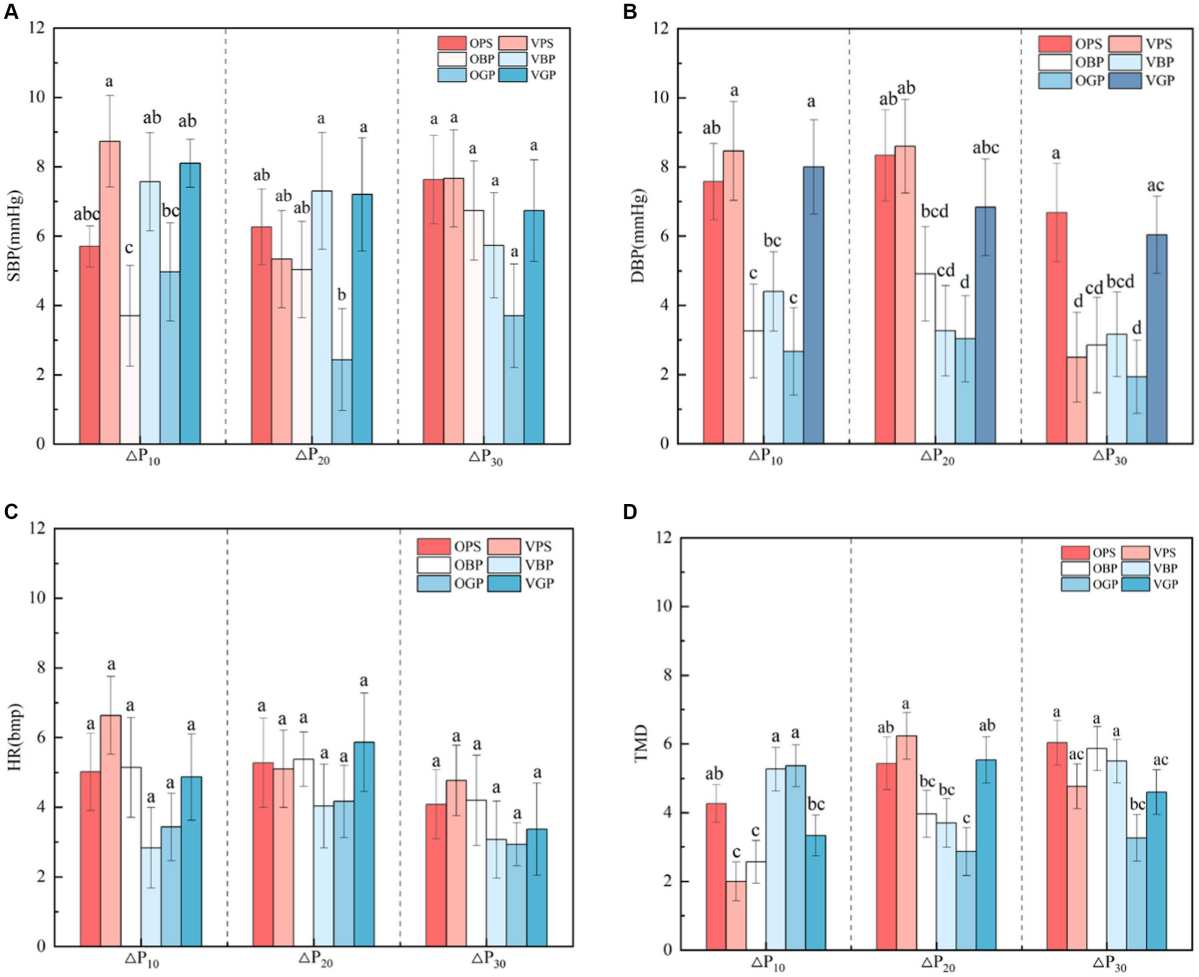
Comparison of differences in physiological and psychological aspects with six scenarios. Recovery amounts of SBP **(A)**, recovery amounts of DBP **(B)**, recovery amounts of HR **(C)**, and recovery amounts of TMD **(D)**.

The SBP change value of visitors after perceiving the forests landscape indicated that the interaction between perception methods and perception durations had a significant effect on the SBP recovery effect of visitors after 10 min (Table 4). By further pairwise comparison, it was found that there were significant differences between OBP and VBP (Table 5). △P_10_ of SBP were: VPS (8.73) > VGP (8.10) > VBP (7.56) > OPS (5.70) > OGP (4.97) > OBP (3.70), which indicated that video perception was more conducive to the rapid recovery of SBP, and the recovery effect of video watching PS was the best (Figure 3).

**Table 4:** Effects of the interaction of perception methods and perception durations on physiological and psychological indicators.

Indicator		OPS	VPS	OBP	VBP	OGP	VGP	*p*
SBP	△P_10_	5.70 ± 3.25	8.73 ± 7.24	3.70 ± 7.96	7.57 ± 7.74	4.97 ± 7.74	8.10 ± 3.80	0.024*
	△P_20_	6.27 ± 5.98	5.33 ± 7.67	5.03 ± 7.60	7.30 ± 9.24	2.43 ± 8.04	7.20 ± 8.94	0.181
	△P_30_	7.63 ± 6.99	7.67 ± 7.66	6.73 ± 7.84	5.73 ± 8.33	3.70 ± 8.18	6.73 ± 8.03	0.374
DBP	△P_10_	7.63 ± 6.02	8.47 ± 7.84	3.27 ± 7.46	4.40 ± 6.30	2.67 ± 6.91	8.00 ± 7.48	0.002**
	△P_20_	8.43 ± 7.28	8.60 ± 7.42	4.97 ± 7.53	3.27 ± 7.13	3.03 ± 6.83	6.83 ± 7.65	0.006**
	△P_30_	6.90 ± 7.81	2.50 ± 7.11	2.87 ± 7.54	3.17 ± 6.69	1.93 ± 5.80	6.03 ± 6.10	0.024*
HR	△P_10_	5.03 ± 6.14	6.63 ± 6.12	5.13 ± 7.85	2.83 ± 6.34	3.43 ± 5.29	4.87 ± 6.79	0.261
	△P_20_	5.33 ± 7.04	5.10 ± 6.08	5.40 ± 4.26	4.03 ± 6.58	4.17 ± 5.68	5.87 ± 7.74	0.849
	△P_30_	4.17 ± 5.48	4.77 ± 5.53	4.20 ± 7.15	3.07 ± 6.06	2.93 ± 3.39	3.37 ± 7.25	0.808
TMD	△P_10_	4.27 ± 0.55	2.00 ± 0.57	2.57 ± 0.62	5.27 ± 0.63	5.37 ± 0.60	3.33 ± 0.60	0.000**
	△P_20_	5.43 ± 0.76	6.23 ± 0.68	3.97 ± 0.68	3.70 ± 0.71	2.87 ± 0.70	5.53 ± 0.67	0.006**
	△P_30_	6.03 ± 0.65	4.77 ± 0.65	5.87 ± 0.64	5.50 ± 0.63	3.27 ± 0.68	4.60 ± 0.65	0.032*

OPS, Onsite perception *Pinus sylvestris*; VPS, Video perception *Pinus sylvestris*; OBP, Onsite perception *Betula platyphylla*; VBP, Video perception *Betula platyphylla*; OGP, Onsite perception Grass plot; VGP, Video perception Grass plot (**p < 0.01, *p < 0.05).

**Table 5:** Pairwise comparison between sites based on the significant effects of the interaction between perception methods and perception durations on SBP, DBP, and TMD.

Indicator		Site	Mean difference	*p*	95% CI of the difference	
					Lower	Upper
SBP	△P_10_	OPS-VPS	−0.07	0.98	−5.51	5.38
	OPS-OBP	−2.93	0.29	−8.38	2.51
	OPS-VBP	3.03	0.27	−2.41	8.48	OPS-OGP	−2.43	0.38	−7.88	3.01	OPS-VGP	0.63	0.82	−4.81	6.08	VPS-OBP	−2.87	0.30	−8.31	2.58	VPS-VBP	3.10	0.26	−2.34	8.54	VPS-OGP	−2.37	0.39	−7.81	3.08	VPS-VGP	0.70	0.80	−4.74	6.14	OBP-VBP	5.98	0.03*	0.52	11.41	OBP-OGP	0.50	0.86	−4.94	5.94	OBP-VGP	3.57	0.20	−1.88	9.01	VBP-OGP	−5.47	0.05	−10.91	−0.02	VBP-VGP	−2.40	0.39	−7.84	3.04	OGP-VGP	3.07	0.27	−2.38	8.51
	DBP	△P10	OPS-VPS	−0.83	0.65	−4.42	2.75	OPS-OBP	4.37	0.02*	0.78	7.95	OPS-VBP	3.23	0.08	−0.35	6.82	OPS-OGP	4.97	0.01**	1.38	8.55	OPS-VGP	−0.37	0.84	−3.95	3.22	VPS-OBP	5.20	0.01**	1.62	8.78	VPS-VBP	4.07	0.07*	0.48	7.65	VPS-OGP	5.80	0.00**	2.22	9.38	VPS-VGP	0.47	0.80	−3.12	4.05	OBP-VBP	−1.13	0.53	−4.72	2.45	OBP-OGP	0.60	0.74	−2.98	4.18	OBP-VGP	−4.73	0.01**	−8.32	−1.15	VBP-OGP	1.73	0.34	−1.85	5.32	VBP-VGP	−3.60	0.05*	−7.18	−0.02	OGP-VGP	−5.33	0.00**	−8.92	−1.75	△P20	OPS-VPS	−0.17	0.93	−3.89	3.56	OPS-OBP	3.47	0.07	−0.26	7.19	OPS-VBP	5.17	0.01**	1.44	8.89	OPS-OGP	5.40	0.01**	1.67	9.13	OPS-VGP	1.60	0.40	−2.13	5.33	VPS-OBP	3.63	0.06	−0.09	7.36	VPS-VBP	5.33	0.01**	1.61	9.06	VPS-OGP	5.57	0.00**	1.84	9.29	VPS-VGP	1.77	0.35	−1.96	5.49	OBP-VBP	1.70	0.37	−2.03	5.43	OBP-OGP	1.93	0.31	−1.79	5.66	OBP-VGP	−1.87	0.32	−5.59	1.86
			VBP-OGP	0.23	0.90	−3.49	3.96	VBP-VGP	−3.57	0.06	−7.29	0.16	OGP-VGP	−3.80	0.05*	−7.53	−0.07	△P30	OPS-VPS	4.40	0.01**	0.90	7.91	OPS-OBP	4.03	0.02*	0.53	7.54	OPS-VBP	3.73	0.04*	0.23	7.24	OPS-OGP	4.97	0.01**	1.46	8.47	OPS-VGP	0.87	0.63	−2.64	4.37	VPS-OBP	−0.37	0.84	−3.87	3.14	VPS-VBP	−0.67	0.71	−4.17	2.84	VPS-OGP	0.57	0.75	−2.94	4.07	VPS-VGP	−3.53	0.05	−7.04	−0.03	OBP-VBP	−0.30	0.87	−3.81	3.21	OBP-OGP	0.93	0.60	−2.57	4.44	OBP-VGP	−3.17	0.08	−6.67	0.34	VBP-OGP	1.23	0.49	−2.27	4.74	VBP-VGP	−2.87	0.11	−6.37	0.64
	TMD	△P10	OPS-VPS	2.27	0.01**	0.60	3.93	OPS-OBP	1.70	0.05*	0.03	3.37	OPS-VBP	−1.00	0.24	−2.67	0.67	OPS-OGP	−1.10	0.19	−2.77	0.57	OPS-VGP	0.93	0.27	−0.73	2.60	VPS-OBP	−0.57	0.50	−2.23	1.10	VPS-VBP	−3.27	0.00**	−4.93	−1.60	VPS-OGP	−3.37	0.00**	−5.03	−1.70	VPS-VGP	−1.33	0.12	−3.00	0.33	OBP-VBP	−2.70	0.00**	−4.37	−1.03	OBP-OGP	−2.80	0.00**	−4.47	−1.13	OBP-VGP	−0.77	0.37	−2.43	0.90	VBP-OGP	−0.10	0.91	−1.77	1.57	VBP-VGP	1.93	0.02*	0.27	3.60	OGP-VGP	2.03	0.02*	−3.70	−0.37	△P20	OPS-VPS	−0.80	0.42	−2.76	1.16	OPS-OBP	1.47	0.14	−0.49	3.43	OPS-VBP	1.73	0.08	−0.23	3.70	OPS-OGP	2.57	0.01**	0.61	4.53	OPS-VGP	−0.10	0.92	−2.06	1.86	VPS-OBP	2.27	0.02*	0.31	4.23	VPS-VBP	2.53	0.01**	0.58	4.49	VPS-OGP	3.37	0.00**	1.41	5.33	VPS-VGP	0.70	0.48	−1.26	2.66	OBP-VBP	0.27	0.79	−1.69	2.23	OBP-OGP	1.10	0.27	−0.86	3.06
			OBP-VGP	−1.57	0.12	−3.53	0.39	VBP-OGP	0.83	0.40	−1.13	2.79	VBP-VGP	−1.83	0.07	−3.79	0.13	OGP-VGP	−2.67	0.01**	−4.63	−0.71	△P30	OPS-VPS	1.27	0.17	−0.54	3.08	OPS-OBP	0.17	0.86	−1.64	1.98	OPS-VBP	0.53	0.56	−1.28	2.34	OPS-OGP	2.77	0.00**	0.96	4.58	OPS-VGP	1.43	0.12	−0.38	3.24	VPS-OBP	−1.10	0.23	−2.91	0.71	VPS-VBP	−0.73	0.43	−2.54	1.08	VPS-OGP	1.50	0.10	−0.31	3.31	VPS-VGP	0.17	0.86	−1.64	1.98	OBP-VBP	0.37	0.69	−1.44	2.18	OBP-OGP	2.60	0.01**	0.79	4.41	OBP-VGP	1.27	0.17	−0.54	3.08	VBP-OGP	2.23	0.02*	0.42	4.04	VBP-VGP	0.90	0.33	−0.91	2.71	OGP-VGP	−1.33	0.15	−3.14	0.48

OPS, Onsite perception *Pinus sylvestris*; VPS, Video perception *Pinus sylvestris*; OBP, Onsite perception *Betula platyphylla*; VBP, Video perception *Betula platyphylla*; OGP, Onsite perception Grass plot; VGP, Video perception Grass plot (**p < 0.01, *p < 0.05).

#### 3.3.2 Change of DBP indicator

By analyzing the DBP change value of visitors after perceiving different forest types on the spot (Table 4), it was found that under the interaction of perception methods and perception durations, the DBP recovery effect of subjects in different forests was significantly different. Further analysis found that based on the different duration of short-term recovery, the DBP change of perceiving of the forests landscape were significant differences between VPS and VBP, OBP, OGP (Table 5). By comparing the amount of recovery found that subjects’ perception of VPS for 10 min and 20 min had the best effect on DBP recovery. The subjects’ perception of OPS for 30 min were significant different from VPS, OBP, VBP, OGP (Table 5). By comparing the amount of recovery found that 30 min perception of OPS had the best effect on DBP recovery. The results showed that VPS is conducive to the short-term recovery of subjects’ DBP, while OPS is more conducive to the long-term recovery of DBP (Figure 3).

#### 3.3.3 Change of HR indicator

The HR changes of visitors after perceiving different forest types, showed that the interaction between perception methods and perception durations had no significant effect on the heart rate recovery effect of visitors (Table 4). This showed that under the same recovery time, there is no significant difference in heart rate recovery between the two forests and grass plot in any way (Figure 3).

#### 3.3.4 Change of TMD indicator

By analyzing the TMD changes of the two pure forests and the grass plot (Table 4), it was found that the interaction between the perception methods and the perception durations had a significant effect on the TMD recovery of the subjects.

Further analysis showed that after the subjects perceived the two pure forests and the grass plot for 10 min, VPS was significant different from OPS, VBP, OGP, and VBP was significant different from OBP, VGP (Table 5). △P_10_ of the OGP was the most obvious. After 20 min of perception of the two forests and the grass plot, the recovery effect of VPS was significant different from VBP, OGP (Table 5). By comparing the amount of recovery (Table 4), the TMD recovery effect of VPS was the best, and the TMD recovery effect of OGP was significantly weakened. After 30 min of perception of the two forests and the grass plot, the TMD recovery effect of OPS and OBP was significant from OGP (Table 5), and the recovery amounts of OPS was the best (Table 4). The results showed that under the interaction of perception methods and perception durations, the influence and difference of pure forests on the recovery of TMD for subjects gradually appear with the increase of recovery time. Video perception showed the advantage of TMD recovery for subjects in a smaller time dose, but onsite perception needs more time for psychological recovery (Figure 3).

In summary, the difference in the pressure relief effect of the two pure forests and grass plot on the human body under the interaction of perception methods and perception durations is mainly reflected in the changes of physiological index DBP and psychological index TMD. The recovery effect of subjects’ DBP is better after 10 min of video perception of two forests, and the video perception grass plot also has a good recovery effect. The recovery advantage of forest for subjects’ DBP began to appear after 20 min of sensing forests, and the recovery effect of VPS was the best. For the TMD index, the recovery effect of video perception reached the best after 20 min, and the recovery effect of onsite perception showed a cumulative effect. With the increase of recovery time, the recovery effect was enhanced. The recovery effect of TMD was better after 20 min of video perception and 30 min of onsite perception.

## 4 Discussion

The purpose of this study is to explore the differences in human stress relief between different pure forests under the interaction of perception methods and perception durations, and to provide scientific basis for the construction of healthy forests and the evaluation of environmental perception preference in the future. Firstly, different forests have different effects on the physiological and psychological health recovery of subjects. PS is a good choice for subjects’ physiological and psychological recovery. Secondly, there is a big difference between the two methods of perception, and video perception is more conducive to the rapid relief of stress. Finally, no matter what kind of perception method is adopted, experiencing different forests under the perception duration of 20 min is more conducive to the physiological and psychological recovery of subjects.

### 4.1 Effects of forests on health recovery

After the visitors perceive the forests landscape, the physiological and psychological indicators show a downward trend, and the physiological and psychological health has been restored to a certain extent. This is consistent with the results of previous studies, which proves that the forest environment is conducive to the visitors to relieve stress (51, 52). At the same time, we also noticed that there were significant differences in the short-term recovery of DBP among subjects. This may be because the recovery of physiological indicators is controlled by parasympathetic nerves, and changes can appear quickly (53). As for the impact of different forests on the recovery effect, the recovery effect of PS is the best, followed by GP, and BP is the worst. It may be because the green environment can relieve people’s tension and anxiety and stabilize emotions, while the large area of white trunks in BP weakens the advantages of the green environment (54). The restoration effect of PS is better than that of GP, probably because people’s landscape preference for trees is higher than that of the grass plot (55). The results also show that there are significant differences in the long-term recovery of visitors’ TMD, which may be since the relief of psychological stress is not controlled by the nervous system, but depends on the mobilization of positive emotions, so the recovery has certain long-term benefits (56). It may also be that the subjects’ preference for green landscape leads to different effects of psychological stress relief, but the recovery advantage of BP for mental health gradually appears with the increase of recovery time. This conclusion is consistent with the previous research results (57).

### 4.2 Effects of perception methods on health recovery

The two perception methods have a positive effect on the physical and mental health of visitors and can relieve stress. This conclusion is in line with previous research results (38, 58). Previous studies have shown that there is no significant difference between non-immersive image perception and onsite perception for stress relief (59). However, this study found that there is a significant difference in the decompression difference between SBP and DBP in the early stage of perception between the two perception methods, and the effect of video perception on different forests is greater than that of onsite perception. This may be due to the fact that perceived short natural video can provide easy-to-obtain low-cost green landscape healing effect, which is helpful to improve physiological and psychological health (60). It may also be because video perception is a single visual stimulus. Onsite perception is the result of visual and other sensory factors, and single stimulation is more direct and rapid than multi-sensory stimulation (21). The long-term recovery of physiological health and the long-term and short-term recovery of psychological health are not different due to different perception methods. This result is consistent with the previous research conclusions. More than a certain length of time, the perception method is no longer the main reason affecting the physical and mental health recovery effect of visitors (61). Therefore, visitors who want to relief from stress quickly, short-term video perception of the forest landscape can provide better healing effects.

### 4.3 Effects of the interaction between perception methods and perception durations on health recovery

#### 4.3.1 Onsite perception in 3 durations

The physiological indications of perceiving different forests and grass plot at 10 min, 20 min, and 30 min were recovered to a certain extent, and the recovery of OPS and OBP showed an upward trend, and the recovery of OGP showed a downward trend. This may be due to the absorption of CO_2_ and the release of O_2_ by photosynthesis of green plants. The secondary metabolites (VOCs) produced in the reaction process can reduce blood pressure and heart rate, while the photosynthesis of trees is higher than that of grass plot. Therefore, the recovery effect of OPS and OBP on physiological indexes is better than that of OGP in three recovery durations (62). For the recovery of SBP and DBP after onsite perception, the recovery of OPS under three different recovery durations showed obvious advantages, and the recovery of SBP at 30 min reached the highest, and the recovery of DBP at 20 min was the best. This may be due to the fact that PS has a denser crown, and the high-density crown can be more fully perceived by subjects, which has a better effect on human stress relief (63). Previous studies have shown that not any length of forest bath has a significant effect on human stress relief, because secondary metabolites need to accumulate to a certain amount to restore physiological health (64). This is consistent with the conclusion of this study. The OPS for 20 min–30 min is more conducive to human physiological health. Subjects onsite perception of different forests and grass plot after 10 min, 20 min, 30 min, the psychological pressure has a certain degree of relief, which is in line with the previous research conclusions, people have subjective expectations for the forest environment, subjective expectations to mobilize the perception of emotions, and thus achieve the effect of psychological healing (65). The recovery difference of the TMD onsite perception of the grass plot for 10 min is higher than that of the forests. This may be due to the fact that although the high-density forest space is private, the psychological security of the strange environment visitors is reduced, and the lookout attribute of the grass plot is magnified (66). Therefore, after people perceive 10 min on the spot, the grass plot can make people freer from tension and anxiety. With the increase of time, the sense of shelter of trees appeared, so the recovery difference of TMD indicator of OPS and OBP was higher than that of OGP.

#### 4.3.2 Video perception in 3 durations

The physiological indexes of 10 min, 20 min, and 30 min of video perception of different forests and grass plot have a certain degree of recovery. For the three indexes of SBP, DBP and HR, the recovery difference of VPS and VGP in different duration is higher than that of VBP. This may be since people appreciate different forests through video in the indoor environment, visual stimulation is the only way of perception, and people’s preference for landscape elements such as green and sky is prominent. Therefore, VPS and VGP are most conducive to the recovery of physiological health (67). With the increase of perception time, the recovery trend of each physiological index decreases. It may be due to long time watching the screen, resulting in physical fatigue. The TMD recovery amounts of different forests and grass plot were restored after 10 min, 20 min, and 30 min of video perception, and the effect of VPS and VGP on relieving psychological pressure was still better than that of VBP. This may be the result of the long-term effect of psychological recovery and the visual preference of visitors, and the psychological recovery also showed a downward trend after 30 min of video perception, indicating that the fatigue caused by video perception was not only visual, but also caused psychological boredom.

## 5 Limitations

This study examined the effects of two pure forests on the physiological and psychological health of the subjects under the interaction of perception methods and perception durations. Although we have made careful preparations, there are still some shortcomings in this study. Firstly, the subjects are college students. The number and age composition of the subjects have limitations. The research results cannot represent all kinds of social groups. In the future, people of different ages, different genders and different health conditions can be further studied. Secondly, there are only two pure forests in the study, and the seasonal changes of forests are not considered. The perceived preference of the subjects may affect the results of the study. Therefore, in the future research, the number of pure forests can be increased, and the experiment can be carried out in different seasons. Finally, future research can also consider adding more sophisticated instruments, such as EEG, skin inductors, etc., to facilitate more scientific and multidimensional display of physiological and psychological recovery effects.

## 6 Conclusion

This study explored the effects of different pure forests on the recovery of people’s physiological and psychological health through different perception methods and different durations of short-term recovery, aiming to provide scientific support for the construction of healthy forests and environmental perception evaluation. The results show that, firstly, the forest environment is conducive to people’s physiological and psychological recovery, and the PS shows obvious recovery advantages. Secondly, onsite perception and video perception have their own advantages in short recovery effect. Video perception is more conducive to the short-term recovery of SBP and DBP. Onsite perception shows potential for the long-term recovery of TMD. With the increase of recovery time, the recovery effect of the two methods tends to be consistent. Finally, based on the perception of forest environment at different duration, DBP and TMD showed significant differences among two pure forests. The recovery of DBP reached the maximum after 20 min of perception of PS in different ways. The perception of OPS showed a cumulative effect, and the recovery amount was the largest at 30 min. The recovery amount of TMD reached the maximum after 20 min of VPS, and gradually lost the advantage of forests with the increase of recovery time. Last not the least, based on the above conclusions, using different perception methods to perceive PS for 20 min has a better recovery effect.

## Data availability statement

The original contributions presented in the study are included in the article/supplementary material, further inquiries can be directed to the corresponding author.

## Author contributions

WN: Conceptualization, Funding acquisition, Methodology, Writing – original draft. QL: Visualization, Writing – original draft. JY: Formal analysis, Writing – review & editing. JH: Investigation, Writing – original draft. YZ: Formal analysis, Writing – original draft. XZ: Resources, Writing – original draft. MS: Data curation, Writing – original draft. XS: Conceptualization, Writing – review & editing.
